# Radiovesicolomics-new approach in medical imaging

**DOI:** 10.3389/fphys.2022.996985

**Published:** 2022-10-10

**Authors:** Ewa Ł. Stępień, Carina Rząca, Paweł Moskal

**Affiliations:** ^1^ Faculty of Physics, Astronomy and Applied Computer Science, M. Smoluchowski Institute of Physics, Jagiellonian University, Kraków, Poland; ^2^ Total-Body Jagiellonian-PET Laboratory, Jagiellonian University, Kraków, Poland; ^3^ Center for Theranostics, Jagiellonian University, Kraków, Poland

**Keywords:** extracellular vesicles, medical imaging, positronium imaging, PET, total-body PET, theranostics

## Abstract

This review introduce extracellular vesicles (EVs) to a molecular imaging field. The idea of modern analyses based on the use of omics studies, using high-throughput methods to characterize the molecular content of a single biological system, vesicolomics seems to be the new approach to collect molecular data about EV content, to find novel biomarkers or therapeutic targets. The use of various imaging techniques, including those based on radionuclides as positron emission tomography (PET) or single photon emission computed tomography (SPECT), combining molecular data on *EVs*, opens up the new space for radiovesicolomics—a new approach to be used in theranostics.

## Introduction

### What is *omics*?

The magic word *omics* appeared in the 1980s as a figment of the imagination and extraordinary creativity of three scientists in the field of genetics: Dr. Thomas H. Roderick (a geneticist at the Jackson Laboratory, Bar Harbor), Dr. Frank Ruddle (Yale University) and Dr. Victor McKusick (The Johns Hopkins University) in 1986. During an international meeting in Kuska (1998) on the feasibility of mapping the entire human genome, they “assembled” a short sub-meeting to discuss starting a new genome-oriented scientific journal. The invention of a new word by T.H. Roderick was the beginning of this vocabulary scientific activity which, like a rush of life-giving water, spilled over all the specialties of molecular biology, yielding crops in the form of all kinds of *omic* fruits (Kuska, 1998). Following genomics, the word *proteomics* was first proposed much later by Marc Wilkins in 1995 to describe the variety of proteins that make up the whole organism (Yadav, 2007). A typical downstream neologism is a portmanteau derived from two words: a core that describes the biological or molecular level of analysis and the suffix *-omics* to denote all studies conducted on a wide scale in a living organism.

**FIGURE 1:**
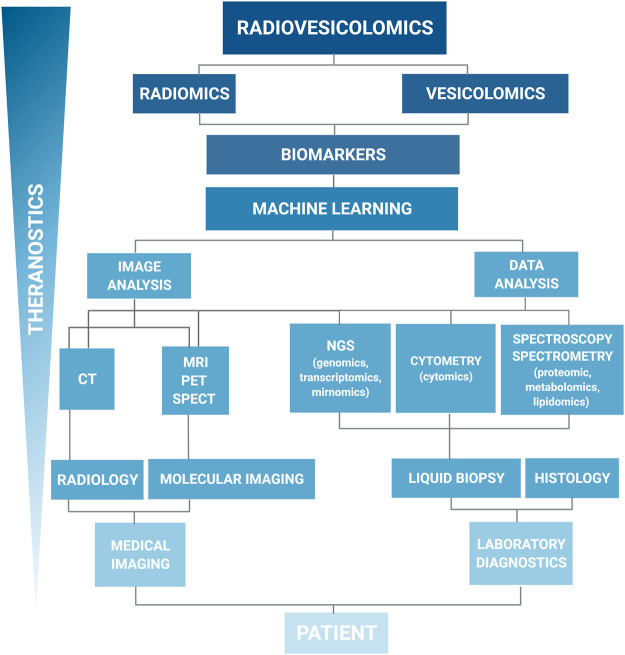
Hierarchical organization of the data workflow for the systemic data collection and analysis. The left branch represents imaging data, the right branch represents the *omic* data. Such organization of the data collection systems is showing the integration and synergy of imaging and laboratory diagnostics for new biomarkers discovery. Liquid biopsy, including extracellular vesicle samples, is an alternative for traditional histology. On the top, radiomics and vesicolomic are coupled to form radiovesicolomics as the new approach in medical imaging and theranostics.

The outstanding development of molecular biology techniques, such as mass spectrometry, molecular sequencing, and chromatographic techniques, caused the creation of many datasets containing quantitative and qualitative characteristics, as the results of large-scale experiments: *genomics*, *transcriptomics*, *metabolomics*, *mirnomics* and *lipidomics* (Skotland et al., 2017; Skotland et al., 2020), as well as *glycomic* studies (Gerlach and Griffin, 2016) (Figure 1). These datasets are stored in repositories as open data sources and are constantly expanded and cured. Great improvements have also been observed in cytometric methods, including flow cytometry techniques, using high-resolution flow cytometry based on the scattering angle analysis and the development of imaging flow cytometry, which is a combination of quantitative flow power with high-quality analysis of large numbers of images. This allowed for the improvement of resolution of flow cytometry for spatial discrimination between nuclear and cytoplasmic fluorescence or cellular morphometry (Pierzchalski et al., 2011).

These new approaches in molecular biology have been transferred to cytometry, a laboratory technique, which development resulted in providing new parameters, complementary to fluorescence intensity, e.g. like fluorescence localization, cell shape and morphology. Translation of new characteristics derived from biochemistry and molecular biology to cellular systems, expressed as the imaging data, together with the strength of “population” statistics, opened up a new window for a novel branch of systemic study—*cytomics* (Valet et al., 2006). *Cytomics* is defined as the multimolecular cytometric analysis of cell and cell system heterogeneity by means of supervised or unsupervised data-mining algorithms. This approach allows for data extraction and effective analysis of multi-parameter data sets in order to obtain the maximum information about the molecular phenotypes of cells (Valet et al., 2006; Valet, 2022)

Another example of this emergence is the formation of the word *radiomics* that currently appeared in the medical literature. Despite some controversies that may result from the connotation of this word, the literature adopts *radiomics* as the application not only radiological examinations, but all imaging tests aimed at comprehensive diagnosis to extract a large number of quantitative features from digital images (Figure 1). The main directions and applications of *radiomics* to personalize the patient treatment have been established by the “Father of Radiomics” Dr Robert (Bob) J. Gillies in his fundamental article (Gillies at al., 2016). The next step in the development of the concept of comprehensive image data analysis is the idea of using data from the whole-body (WB) images—*imiomics* (Strand et al., 2017). The concept of *imiomics* derived from the holistic Magnetic Resonance Imaging (MRI) data analysis, where the information in each voxel is collected from a patient to compare between patients or analyzed over time and integrated with other *omics* data within a patient to visualized fat tissue distribution (Lind et al., 2019).

In our scheme, in an equal level of this creative terminology, we have placed the second term, created to draw attention to the comprehensiveness and emergence of research into new biomarkers: extracellular vesicles (*EVs*)—*vesicolomics*. Starting from this article, we would like to apply the *vesicolomics* as a new concept to characterize *EVs* and collect data to extract maximal information from standard and high-throughput methods used in *EV* research.

### What *omics* can do for theranostics?

Theranostics is a portmanteau word derived from terms therapeutic (Greek *therapeia*) and diagnostics (Greek *diagnõsis*). Without an accurate diagnosis, based on laboratory or imaging tests, an appropriate treatment cannot be applied. Theranostics means the use of molecular and functional imaging to determine the location, size and type of a lesion. It can be a neoplastic lesion, but also other types of lesions, such as an enlarged thyroid gland, prostate gland, pituitary gland, inflammatory lesion and the like (Opalińska et al., 2021). If the marker for the lesion localization is a radioisotope (radioactive element), diagnostics consists in measuring the signal (radiation), and analyzing the distribution of the radiation signal in four dimensions: 3D in space and a time dimension. The simultaneous use of radioactive isotopes for diagnosis also enables radiotherapy, which may be used alone or in combination with other treatments for the individual patient (Weineisen, et al., 2015; Królicki and Kunikowska, 2021). *Omics* is an approach to the foundation of new biomarkers that allows objective extraction and selection of new therapeutic and diagnostic targets based on the analysis of clinical and experimental (genetic, biochemical, histological and imaging) data (Gillies et al., 2016; Wróbel 2021).

The new therapeutic targets are very important for theranostics, there is limited number of theranostic radionuclides (Choiński and Łyczko, 2021; Matulewicz 2021), but the number of theranostic biomarkers and targets is endless.

## Extracellular vesicles—new objects for *omics*

### Extracellular vesicles definition and classification

**FIGURE 2:**
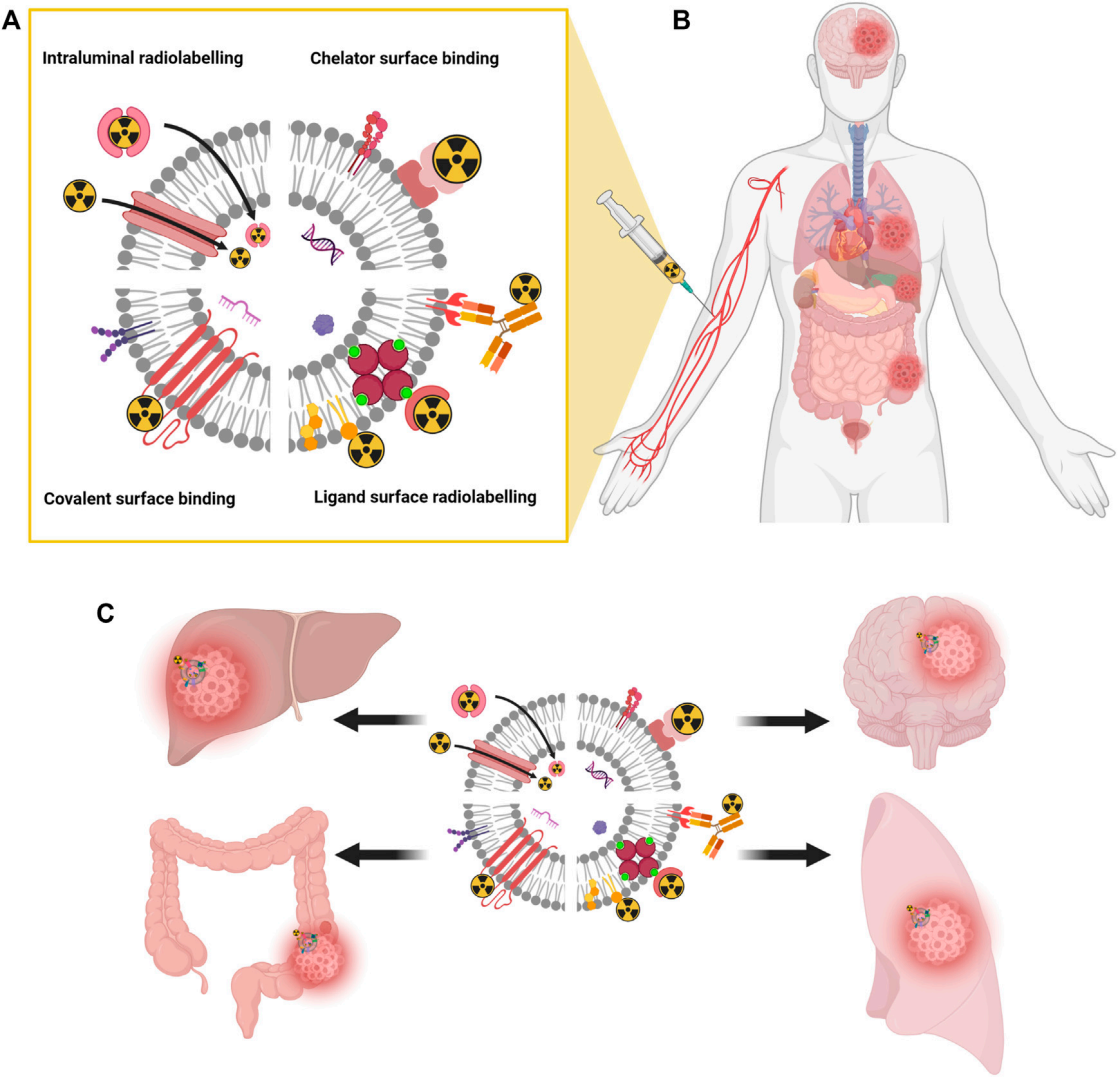
Schematic representation of different EVs radiolabeling methods. Surface radiolabeling, covalent binding, and intraluminal radiolabeling **(A)**. Radiolabeled EVs injected into a patient bloodstream **(B)** delivers radiation therapy and drugs specifically and directly to disease sites **(C)**. Created with BioRender.com.

*EVs* are nano- and micro-sized double-layered membrane entities produced by most cell types and released into biological fluids enabling cell-to-cell communication at close or distant sites (Kim et al., 2014). These nano- and microfragments of cell membranes are classified according to their formation and differences in size (diameter), into subgroups, including exosomes (Ex) with a diameter of 30–100 nm, ectosomes with a diameter of 100 nm to 1 μm (Figure 2), and apoptotic bodies (AB) with a size between 1 and 5 μm (Gurunathan et al., 2021). A single cell can release different types of *EVs*, resulting their heterogeneity within the *EVs* subtype. Currently, the recommendations of the International Society of Extracellular vesicles (ISEV), namely the 2018 MISEV guidelines, endorsed the use of the term “extracellular vesicles” (small, medium or large) instead of e.g. exosome, ectosome *etc.* (Théry et al., 2018).

### Vesicolomics as the multimodal approach for *EVs* characterization

The dynamic development of cell biology and, above all, the interest of biologists and biophysicists in *EVs*, resulted in offering new research tools and techniques used in material sciences and their application in research at the subcellular and nanoscale levels, e.g. atomic force microscopy (Gajos et al., 2017; Stępień et al., 2018) infrared Attenuated Total Reflectance (ATR) spectroscopy (Stępień et al., 2018; Paolini et al., 2020), dynamic light scattering (DLS) (van der Pool et al., 2013) and tunable resistive pulse sensing (van der Pol et al., 2013). Such analyses, combined with results of high-throughput techniques, produce a vast number of multiparametric (quantitative and qualitative) data from less number of examined samples. It needs systematic and multimodal analyses for integration of *omics* datasets and selection highly correlated biological features using different bioinformatics methods like Canonical Correlation Analysis (CCA) (Jun et al., 2018; Turek et al., 2020; Wróbel 2021) or deep/machine learning algorithms for better selection of biological interrelationships (Stahlschmidt et al., 2022).

### *EVs* as biomarkers for liquid biopsy

The rapid expansion of molecular biology techniques, including high-throughput genetic techniques such as DNA and RNA sequencing (next generation sequencing—NGS) (Cheng et al., 2014; San Lucas et al., 2016), as well as mass spectroscopy techniques in *proteomic*, *lipidomic*, *metabolomic* and *glycomic* (Williams et al., 2018) studies, has created new perspectives for offering extracellular vesicles as biomarkers in different pathologies. Circulation *EVs* are present in all body fluids to offer a high level of sensitivity and specificity of the non-invasive medical procedure through the collection of body fluid samples such as blood or urine (Stępień et al., 2012; Stępień et al., 2020). *EVs* were proposed as biomarkers in such diseases as diabetes (Tokarz et al., 2015, 2019; Alexandru et al., 2016; Stępień et al., 2018; Kamińska et al., 2022; Roman et al., 2019), cardiovascular diseases including stroke (Lee et al., 1993; Lundström at al., 2020), myocardial infarction (Stępień et al., 2012; Burrello et al., 2020), atherosclerosis and stable coronary artery disease (Chyrchel et al., 2019; Gkaliagkousi et al., 2021). The uniqueness of *EVs* in terms of their molecular composition has become a great promise to be explored as a new source of biomarkers in liquid biopsy, which was recognized in cancer diagnostics (Surman et al., 2018; Möller and Lobb, 2020; Stępień et al., 2021a).

### Immune system and the dynamics of *EV* biodistribution

The average plasma half-life of intravenously delivered *EVs* is reported to be between 30 and 80 min (Lai, et al., 2014; Charoenviriyakul et al., 2018) (Figure 2B). It is due to the phagocyting activity of mononuclear phagocyte system (MPS), which is generally involved in biodistribution, organ accumulation and a half-life of *EVs.* Most of the intravenously delivered *EVs* are internalized and transported by phagocyting cells and ultimately accumulate in the spleen, lungs, liver, and gastrointestinal tracts (Matsumoto, et al., 2017; Charoenviriyakul et al., 2018) (Figure 2C). In such targeting organs *EVs* have the preference to endothelial and Kupffer cells (Kooijmans et al., 2016; Németh et al., 2021). The *EV* half-life can be potentially increased by the reduction of cationic α-d-mannose monosaccharide or phosphatidylserine exposure (Escrevente et al., 2011; Matsumoto et al., 2017). Such modifications of *EVs* to control their uptake and biodistribution are called “eat me/do not eat me” strategy to achieve effective drug delivery: MPS saturation (eat me) to increase dendritic cells stimulation or *EV* uptake by cancer cells or avoid phagocytosis and increase organ targeting (do not eat me) (Escrevente et al., 2011; Choi et al., 2019; Belhadj et al., 2020).

Interestingly, the cellular origin of *EVs* also influence their distribution, suggesting that *EVs* from different cellular sources have different targeting properties. In order for *EVs* to perform their function, they must first bind to the target cell, and it is known that different *EVs* are able to preferentially bind to specific target cell types. This innate ability of *EVs* to bind to target cells is a feature that can be exploited to target *EV* drug carriers to the desired sites of action.

## Extracellular vesicles as drug delivery systems

In addition to the outstanding and unique diagnostic applications of *EVs* as a liquid biopsy, the other virtually limitless potential of *EVs* is seen, as possible drug delivery systems (DDS) in many diseases (van Dommelen, et al., 2012; Hwang et al., 2015; Kojima, et al., 2018). Exosomes as nanoscale membrane vesicles with a special ability to target specific cells may serve as carriers to mediate a horizontal gene transfer. This potential has been firstly recognized as the transfer of mRNA or short, non-coding RNA (micro RNA-miRNA) in health and diseases (Baj-Krzyworzeka et al., 2006; van Dommelen et al., 2012) to set new directions for research on the biomimetic properties of *EVs*. Currently, loading of *EVs* with exogenous miRNA or pre-miRNA is a tempting strategy to achieve the antitumor effect of *EVs* (Ohno et al., 2013; Sutaria et al., 2017).

The most important characteristic to nominate *EVs* as candidates for contemporary DDS are as follows: biological stability, cell targeting, plasma protein interactions (pharmacodynamics) and controlled drug release.

### Biological stability and lifespan of extracellular vesicles

*EVs* are continuously released parental cell and uptaken by target cells, which can be distant significant, thus it is impractical to evaluate the lifespan of an average vesicle, and such information is still missing. The EV stability and shelf life in biological fluids is better described. The best conditions for *EV* storage is freezing. The small *EVs* (exosomes) preserve their size and protein content at -80°C for 28 days, showing comparable biodistribution to freshly isolated ones (Wu et al., 2021). Also freezing temperatures preserved most *EV* particles, and 4°C and 20°C would cause significant loss of *EV*s (Lőrincz et al., 2014). Biological activity of exosomes is significantly increase after addition of trehalose to improve their long-term stability (Bosch, et al., 2016; Charoenviriyakul et al., 2018). In contrast, dendritic cells-derived *EVs* are stable and can be stored frozen for at least 6 months (Alvarez-Erviti et al., 2011). Another way to enhance EV stability is a modification by polyethylene glycol (PEGylation) to achieve better blood residence and cell targeting (Kooijmans et al., 2016; Shi, et al., 2019).

### Cell targeting by extracellular vesicles

The pivotal feature of *EV*-based DDS is their targeting capacity, which is limited by two physiological boundaries: a vascular barrier and a target recognition. Systemic administration via intra venous injection of *EVs* is not effective when target cells are located distantly (organized tumor) or when delivery is to the brain. In such case the direct administration to the organ or by peritoneal injection is a method to improve cell targeting (Wiklander et al., 2015; Chen et al., 2021). The other method is to tag of *EVs* with a signaling peptide or ligand to improve targeting (Alvarez-Erviti et al., 2011). The generation of tagged *EVs* through transfection with ligand expression vectors for lactadherin or Rabies Viral Glycoprotein (RVG) peptides is a proposed protocol for a direct cell targeting (Alvarez-Erviti et al., 2011; Matsumoto et al., 2017; Kojima et al., 2018). Lamp2b, and tetraspanins could serve as a promising strategy for active targeting of cancer cells for therapeutic exosomes (Kojima et al., 2018; Wang, 2022). The other strategy is to produce tumor *EVs* (*Tu-EVs*), which have a natural housing behavior to target cells (Lara, et al., 2020; Gong et al., 2021) or are used as a tumor antigens-adjuvant utilizing *Tu-EVs* as a tumor cell-based vaccine to target dendritic cells (DCs) (Huang et al., 2022). *EVs* from HEK293T cells accumulated in subcutaneous tumors, which may be exploited by *EV*-based anticancer therapies (Murphy et al., 2019).

“Eat me” strategy can be also used to improve cell targeting by altering the *EV* glycation pattern (Escrevente et al., 2011; Choi et al., 2019) (see *Cell targeting by extracellular vesicles*). Very promising approach to *EV* targeting is using *EVs* derived from immune cells: macrophages or dendritic cells (DCs) to target inflammatory sites and regulate the inflammatory response. This strategy is applied to deliver therapeutic agent directly to neural cells, brain tumors (Alvarez-Erviti et al., 2011; Liang et al., 2021) or affect antitumor immune responses (Fernández-Delgado et al., 2020).

### Passing the brain-blood barrier by *EVs* to achieve therapeutic effect in glioma

Several types of circulating *EVs* interact with brain microvascular endothelial cells and modulate the integrity of the brain-blood barrier (*BBB*), e.g. glioma-derived EVs can pass the intact BBB and are detected in the peripheral blood of patients (García-Romero et al., 2017). This process is controlled by various mechanisms among them inflammation being the larger contributor. A similar mechanism can be used by DDD to target glioma cell in the brain enhanced by adoptive transcytosis to enter the central nervous system parenchyma (Krämer-Albers et al., 2022). To deliver drugs to brain tumors, peptide-modified *EVs* need to be generated to pass the BBB and targeted glioma. The best results are observed for a peptide targeting low-density lipoprotein receptor-related protein-1 (LRP1), which mediates the transcytosis across the BBB, such as Angiopep-2 peptide or the integrin family protein - leukocyte function associated antigen 1 (LFA-1) (Shan et al., 2022; Zhu et al., 2022). The other strategy may apply tumor derived *EVs* as a potential glioma vaccination due to their ability to display tumor antigens that can activate DCs, which can then activate CD8^+^ T cells having antitumor potential (Fernández-Delgado et al., 2020).

## Systems radiomics and extracellular vesicles tracking

Radiomics can be performed with tomographic images obtained from computed tomography (CT), magnetic resonance imaging (MRI) or positron emission tomography (PET) (Gillies et al., 2016). The most sensitive imaging modality is PET (Alavi et al., 2021), allowing to detect 10^−11^–10^−12^ M concentrations of radiolabeled agent, which is an equivalent of nanograms for an injection to a human body (Skotland et al., 2022). Single photon emission computed tomography (SPECT) is more convenient because of availability of radionuclides and detectors, nevertheless the sensitivity of this technique is one order of magnitude lower then PET (Skotland et al., 2022). In preclinical (animal) studies, the most common modalities are optical and fluorescence techniques utilizing lipophilic fluorescence dyes (Kojima et al., 2018; Wu et al., 2021). The greatest advantage of fluorescence markers is their accessibility and utility, the disadvantage is the low sensitivity falling to the concentration of fluorescent agent in the range of 10^−9^–10^−11^ M and the limit for depth detection between 1 and 10 cm (Lázaro-Ibáñez et al., 2021; Skotland et al., 2022). These EV imaging approaches are used in preclinical studies and needs the translation to clinical practice (Arifin et al., 2022).

### Strategies for radiolabeling of *EVs*

Since extracellular vesicles are the cell-derived structures enveloped by a lipid bilayer, which is like a typical biological membrane, the strategies for *EV* labeling are almost identical with those for cell labelling (Gawne et al., 2022). Consequently, *EVs* can be radiolabeled by surface tagging or intraluminal loading methods (Figure 2A). In the surface radiolabeling, radionuclide can be a part of a stable radiopharmaceutical (antibody or ligand) whish recognize a specific antigen or receptor on a *EV* surface (Morishita et al., 2015). Alternatively, a radiopharmaceutical can be directly incorporated to a lipid membrane. In the covalent bounding strategy, a useful chelator for various radioisotopes (e.g. NOTA) can be conjugated with the amine group of the membrane proteins on EVs (Royo et al., 2019; Shi et al., 2019; Jung et al., 2020; Lázaro-Ibáñez et al., 2021). In the intraluminal radiolabeling strategy, a lipophilic radiotracer can easily penetrate to the *EV* lumen or ionophores allow radionuclides to be transported across the lipid membrane where they can be trapped as their loose lipophilicity (Son et al., 2020; Khan et al., 2022).

### *In vivo* tracking of *EVs*

Current studies demonstrated that radiolabeling is the most sensitive *EV* tracking approach for a quantitative biodistribution and pharmacokinetic study (Lázaro-Ibáñez et al., 2021). *EVs* radiolabeled with a bifunctional chelator as diethylenetriaminepentaacetic acid (DTPA) and the indium trivalent isotope ([^111^In]-DTPA) were detected in BALB/c tumor-bearing mice at a dose of 10^11^ vesicles administered *i. v.* (Lázaro-Ibáñez et al., 2021). With its radioactive decay half-life (*t*_*1/2*_) of 2.83 days, the ^111^In isotope is an appropriate for *EVs* imaging studies that extend over several days. The alternative isotope is technetium (^99m^Tc) with its radioactive *t*_*1/2*_ of 6 h. The advantages of ^99m^Tc radiolabeling an easy preparation of a radiopharmaceutical before its administration and the emission of a monochromatic γ radiation (140.5 keV, 98.6%) make this radioisotope achievable for different preclinical and clinical studies. For both radionuclides, SPECT and SPECT/CT are imaging modalities in biodistribution studies (Hwang et al., 2015; Lázaro-Ibáñez et al., 2021). However, for the *in vivo* EV tracking, ^99m^Tc radiolabeling appears to be inefficient due to its short decay half-life *t*_*1/2*_. Using this radiotracer, the uptake of red blood cell-derived exosome-mimetic vesicles (^99m^Tc-RBC-EMVs) was shown to be dose- and time-dependent reaching its maximum at 12–18 h of incubation, too long to be clinically applicable (Son et al., 2020).

The *in vivo* PET imaging of *EVs* is achievable by the gallium ^68^Ga (*t*_*1/2*_ = 68 min), cooper ^64^Cu (*t*_*1/2*_ = 12.7 h), zirconium ^89^Zr (*t*_*1/2*_ = 78.4 h) and iodine ^124^I (*t*_*1/2*_ = 100 h) isotopes, with the increasing half-life (Royo et al., 2019; Jung et al., 2020; Khan et al., 2022). The advent of high sensitivity total-body PET scanners opens possibility for an efficient *in vivo* tracking of EVs in the whole human body simultaneously (Stępień et al., 2021b; Vandenberghe et al., 2020) (Figure 2C). The long half-life of mentioned radionuclides are ideal for long term *in vivo* tracking of *EVs*, but for the practical achievability of a radionuclide decides its chemical properties and the availability of a biological ligand, which determines radiolabeling conditions (Khan and de Rosales, 2021).

## Radiovesicolomics—Applications of *EVs* in nuclear imaging

Nuclear imaging, especially PET, in the extremely developing imaging modality having the most realistic perspective to be used in radiovesicolomics. The main advantage of nuclear imaging is ability to obtain 3D whole body images (Khan and de Rosales, 2021; Lázaro-Ibáñez et al., 2021). Notably, recently a new generation of PET scanners was introduced enabling dynamic and kinetic model based imaging of all tissues and organs simultaneously (Badawi et al., 2019; Karp et al., 2020; Moskal et al., 2021c; Moskal and Stępień, E. Ł. 2020; Prenosil et al., 2022; Spencer et al., 2021). Another advantage of SPECT or PET imaging modalities is their very high sensitivity reaching a factor 10^−6^ with compare to MRI. This allows application of the radionuclide in a dose from very small dose 0.2–1 MBq per mouse for whole-body imaging with the use of EVs in a concentration 10^10^ of particles/Gram body weight (p/g) (Lázaro-Ibáñez et al., 2021; Khan et al., 2022). Depending on imaging radionuclides and the detection system, the applied dose can vary from 37 kBq (^125^I-biotin) (Matsumoto et al., 2017) to 5–10 MBq ([^111^In]DTPA) (Lázaro-Ibáñez et al., 2021), 7 MBq (^64^Cu-NOTA) (Banerjee et al., 2019), 3.7 MBq (^99m^Tc) (Son et al., 2020), 2 MBq (^64^Cu-NOTA) (Shi et al., 2019), and 0.2–1 ^89^Zr-PANC1 (Khan et al., 2022) to track/image EVs using either SPECT or PET in a mouse. Radiovesicolomics may benefit also from the new multi-photon PET scanners (Moskal et al., 2020; Moskal et al., 2021a; Moskal et al., 2021b; Moskal et al., 2021c) which enable simultaneous multi-tracer imaging (Moskal and Stępień, 2022) and hence studying simultaneously the kinetics of two different types of EVs by marking them with different isotopes.

### Radiovesicolomics in cardiovascular diseases

**FIGURE 3:**
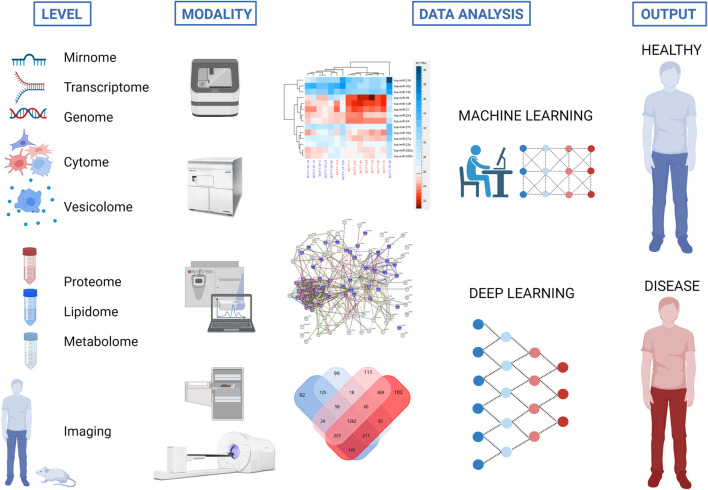
Strategy to use different *omics* and imaging modalities for characterization of extracellular vesicles (*EVs*) to define new biomarkers and therapeutic targets for disease recognition and treatment. The same tools for proteome, mirnome, genome, transcriptome, lipidome and metabolome analyses may serve for *EV* characterization (vesicolome). Created with BioRender.com.

Regenerative medicine is the promising perspective for use of *EVs* in cardiovascular Theranostics (Gąsecka et al., 2018), Recent studies have shown that *EVs* exhibit various regenerative properties valuable in the treatment of cardiovascular disease. *EVs* derived from bone marrow mesenchymal stem cells improve cardiac function and promote angiogenesis in acute myocardial infarction (AMI) (Wang et al., 2017). These pro-angiogenic potential arises from paracrine effectors regulated by NF-kB signaling including platelet-derived growth factor (PDGF), epidermal growth factor (EGF), and fibroblast growth factor (FGF) identified both in endothelial and stem cell derived EVs (Tokarz et al., 2015; Anderson et al., 2016). Another possible mechanism is epigenetic regulation via miRNA carried by *EVs* which may have both pro- and anti-angiogenic activity (Bei et al., 2017; Stępień et al., 2018; Wang et al., 2017). The transfer of these bioactive molecules is possible due to the internalization of *EVs* by the recipient angiogenic cells (Durak-Kozica et al., 2018). In this case, radiovesicolomics proposes the use of imaging studies, with the use of radionuclide-labeled *EVs*. This approach will allow the precise location and distribution of *EVs* having an angiogenic potential in the treatment of heart failure caused by hypoxia or inflammation (Figure 3).

### Radiovesicolomics in diabetes

Global diabetes mellitus prevalence in the population ages 20 to 79 reached 9.8% in 2021. Among many complications caused by diabetes, microvascular complications are the most common, typically including retinopathy, nephropathy, and neuropathy, and contribute to increased treatment costs. To lower the cost of treating diabetic complications, new and sensitive biomarkers are needed to accelerate the diagnosis process and allow for the elimination of diabetic complications at the early stage of the disease. To this end, *EVs* appear to be an ideal biomarker demonstrating their characteristic molecular profile, including miRNA, proteins, lipids and metabolites (Tataruch-Weinert et al., 2016; Kamińska et al., 2021; Kamińska et al., 2022) (Figure 3). There is lack of imaging studies using *EVs* in the course of diabetes as a carrier of radionuclides and the development of safe and efficacious delivery strategies for *EVs* in diabetes therapies is still the unmet need in theranostics (Li et al., 2022).

### Radiovesicolomics in cancer

The concept of *omics* and theranostics is related to personalization of treatment, which is of greatest importance in terms of neoplastic disease. What is most important in personalized medicine is the development of a treatment procedure tailored to the patient’s condition, his genetic predispositions (which are mainly related to the metabolic capacity of the drug) and the production of a drug targeted at a given type of cancer. The deep characteristic of *EVs* content in cancer is giving the opportunity to find new biomarkers (shedding cancer proteins, metabolites, miRNAs) related with cancer development and metastasis, or recognizing the new metabolic targets (Surman et al., 2018; Möller and Lobb, 2020; Stępień et al., 2021a). Using the example of overall glioma, *EVs* as the entities passing the BBB barrier, can serve as prognostic and therapeutics approaches (García-Romero et al., 2017; Krämer-Albers, 2022). Radiovesicolomis will help to develop strategies for tumor *EVs* (*Tu-EVs*) labelling and use them for recognition of cancer location or monitoring cancer interaction with other cells e.g. immune cells, which are modulated by *Tu-EVs*.

## Conclusion

This review is the first proposal of radiovesicolomic approaches in theranostics. Undoubtedly, the results of the studies gathered in this manuscript demonstrate that manufacturing, radiolabeling and administering *EVs* is feasible and safe, but there are still some limitations as the unknown lifespan of an average *EV*, the availability of detection systems and unknown mechanism of EV accumulation. The advantages of radionuclide-based imaging modalities make them a promising tool to validate the use of *EVs* in clinical setting, as they have a potential to characterize *in vivo* the pharmacokinetics and biological behavior of extracellular vesicles. Although PET offers better quantification two- or even 3-fold higher sensitivity than SPECT, the latter is still the most widely used imaging technology due to cost-effectiveness, availability and the existence of a wider range of suitable radionuclides. The strategies described here demonstrate how molecular imaging can be useful in guiding the development of biomedical applications of *EVs* for medical diagnosis and treatment in the ever-evolving field of nanotechnology and theranostics.

## References

[B1] Alavi A. Werner T. J. Stępień E. Ł. Moskal P. (2021). Unparalleled and revolutionary impact of PET imaging on research and day to day practice of medicine. Bio-Algorithms Med-Systems 17 (4), 203–212. 10.1515/bams-2021-0186

[B2] Alexandru N. Badila E. Weiss E. Cochior D. Stępień E. Georgescu A. (2016). Vascular complications in diabetes: Microparticles and microparticle associated microRNAs as active players. Bioch. Biophysi. Res. Com. 472 (1), 1–10. 10.1016/j.bbrc.2016.02.038 26891868

[B3] Alvarez-Erviti L. Seow Y. Yin H. Betts C. Lakhal S. Wood M. J. (2011). Delivery of siRNA to the mouse brain by systemic injection of targeted exosomes. Nat. Biotech. 29 (4), 341–345. 10.1038/nbt.1807 21423189

[B4] Anderson J. D. Johansson H. J. Graham C. S. Vesterlund M. Pham M. T. Bramlett C. S. (2016). Comprehensive proteomic analysis of mesenchymal stem cell exosomes reveals modulation of angiogenesis via nuclear factor-KappaB signaling. Stem cells Dayt. Ohio) 34 (3), 601–613. 10.1002/stem.2298 PMC5785927 26782178

[B5] Arifin D. R. Witwer K. W. Bulte J. (2022). Non-Invasive imaging of extracellular vesicles: Quo vaditis *in vivo*? J. Extracell. Vesicles. 11 (7), e12241. 10.1002/jev2.12241 35844061 PMC9289215

[B6] Badawi R. D. Shi H. Hu P. Chen S. Xu T. Price P. M. (2019). First human imaging studies with the EXPLORER total-body PET scanner. J. Nucl. Med. 60, 299. 10.2967/jnumed.119.226498 30733314 PMC6424228

[B7] Baj-Krzyworzeka M. Szatanek R. Weglarczyk K. Baran J. Urbanowicz B. Brański P. (2006). Tumour-derived microvesicles carry several surface determinants and mRNA of tumour cells and transfer some of these determinants to monocytes. Cancer Immunol. Immunother. CII 55 (7), 808–818. 10.1007/s00262-005-0075-9 16283305 PMC11030663

[B8] Banerjee A. Alves V. Rondão T. Sereno J. Neves Â. Lino M. (2019). A positron-emission tomography (PET)/magnetic resonance imaging (MRI) platform to track *in vivo* small extracellular vesicles. Nanoscale 11 (28), 13243–13248. 10.1039/c9nr02512j 31290510

[B111] Bei Y. Tao L. Cretoiu D. Cretoiu S. M. Xiao J. (2017). MicroRNAs mediate beneficial effects of exercise in heart. Adv. Exp. Med. Biol. 1000, 261–280. 10.1007/978-981-10-4304-8_15 29098626

[B9] Belhadj Z. He B. Deng H. Song S. Zhang H. Wang X. (2020). A combined "eat me/don't eat me" strategy based on extracellular vesicles for anticancer nanomedicine. J. Extracell. Vesicles. 9 (1), 1806444. 10.1080/20013078.2020.1806444 32944191 PMC7480498

[B10] Bosch S. de Beaurepaire L. Allard M. Mosser M. Heichette C. Chrétien D. (2016). Trehalose prevents aggregation of exosomes and cryodamage. Sci. Rep. 6, 36162. 10.1038/srep36162 27824088 PMC5099918

[B11] Burrello J. Bolis S. Balbi C. Burrello A. Provasi E. Caporali E. (2020). An extracellular vesicle epitope profile is associated with acute myocardial infarction. J. Cell Mol. Med. 24 (17), 9945–9957. 10.1111/jcmm.15594 32666618 PMC7520329

[B12] Charoenviriyakul C. Takahashi Y. Nishikawa M. Takakura Y. (2018). Preservation of exosomes at room temperature using lyophilization. Int. J. Pharm. 553 (1–2), 1–7. 10.1016/j.ijpharm.2018.10.032 30316791

[B112] Chen P. Wang L. Fan X. Ning X. Yu B. Ou C. (2021). Targeted delivery of extracellular vesicles in heart injury. Theranostics 11 (5), 2263–2277. 10.7150/thno.51571 33500724 PMC7797669

[B13] Cheng L. Sun X. Scicluna B. J. Coleman B. M. Hill A. F. (2014). Characterization and deep sequencing analysis of exosomal and non-exosomal miRNA in human urine. Kidney Intern 86 (2), 433–444. 10.1038/ki.2013.502 24352158

[B14] Choi E. S. Song J. Kang Y. Y. Mok H. (2019). Mannose-modified serum exosomes for the elevated uptake to murine dendritic cells and lymphatic accumulation. Macromol. Biosci. 19 (7), e1900042. 10.1002/mabi.201900042 31141293

[B15] Choiński J. Łyczko M. (2021). Prospects for the production of radioisotopes and radiobioconjugates for Theranostics. Bio-Algorithms Med-Systems. 17 (4), 241–257. 10.1515/bams-2021-0136

[B16] Chyrchel B. Drożdż A. Długosz D. Stępień E. Ł. Surdacki A. (2019). Platelet reactivity and circulating platelet-derived microvesicles are differently affected by P2Y12 receptor antagonists. Int. J. Mol. Sci. 16 (2), 264–275. 10.7150/ijms.28580 PMC6367525 30745807

[B18] Durak-Kozica M. Baster Z. Kubat K. Stępień E. (2018). 3D visualization of extracellular vesicle uptake by endothelial cells. Cell. Mol. Biol. Lett. 23, 57. 10.1186/s11658-018-0123-z 30574165 PMC6296015

[B19] Escrevente C. Keller S. Altevogt P. Costa J. (2011). Interaction and uptake of exosomes by ovarian cancer cells. BMC cancer 11, 108. 10.1186/1471-2407-11-108 21439085 PMC3072949

[B20] Fernández-Delgado I. Calzada-Fraile D. Sánchez-Madrid F. (2020). Immune regulation by dendritic cell extracellular vesicles in cancer immunotherapy and vaccines. Cancers 12 (12), 3558. 10.3390/cancers12123558 PMC7761478 33260499

[B21] Gajos K. Kamińska A. Awsiuk K. Bajor A. Gruszczyński K. Pawlak A. (2017). Immobilization and detection of platelet-derived extracellular vesicles on functionalized silicon substrate: Cytometric and spectrometric approach. Anal. Bioanal. Chem. 409 (4), 1109–1119. 10.1007/s00216-016-0036-5 27822644 PMC5258792

[B22] García-Romero N. Carrión-Navarro J. Esteban-Rubio S. Lázaro-Ibáñez E. Peris-Celda M. Alonso M. M. (2017). DNA sequences within glioma-derived extracellular vesicles can cross the intact blood-brain barrier and be detected in peripheral blood of patients. Oncotarget 8 (1), 1416–1428. 10.18632/oncotarget.13635 27902458 PMC5352065

[B23] Gąsecka A. van der Pol E. Nieuwland R. Stępień E. (2018). Extracellular vesicles in post-infarct ventricular remodelling. Kardiologia Pol. 76 (1), 69–76. 10.5603/KP.a2017.0178 28980299

[B24] Gawne P. J. Man F. Blower P. J. T M de Rosales R. (2022). Direct cell radiolabeling for *in vivo* cell tracking with PET and SPECT imaging. Chem. Rev. 122 (11), 10266–10318. 10.1021/acs.chemrev.1c00767 35549242 PMC9185691

[B25] Gerlach J. Q. Griffin M. D. (2016). Getting to know the extracellular vesicle glycome. Mol. Biosyst. 12 (4), 1071–1081. 10.1039/c5mb00835b 26888195

[B26] Gillies R. J. Kinahan P. E. Hricak H. (2016). Radiomics: Images are more than pictures, they are data. Radiology 278 (2), 563–577. 10.1148/radiol.2015151169 26579733 PMC4734157

[B27] Gkaliagkousi E. Gavriilaki E. Yiannaki E. Vasileiadis I. Nikolaidou B. Lazaridis A. (2021). Platelet microvesicles are associated with the severity of coronary artery disease: Comparison between peripheral and coronary circulation. J. Thromb. Thrombolysis 51 (4), 1138–1143. 10.1007/s11239-020-02302-5 33043416

[B28] Gong C. Zhang X. Shi M. Li F. Wang S. Wang Y. (2021). Tumor exosomes reprogrammed by low pH are efficient targeting vehicles for smart drug delivery and personalized therapy against their homologous tumor. Adv. Sci. (Weinheim, Baden-Wurttemberg, Ger. 8 (10), 2002787. 10.1002/advs.202002787 PMC8132050 34026432

[B29] Gurunathan S. Kang M. H. Qasim M. Khan K. Kim J. H. (2021). Biogenesis, membrane trafficking, functions, and next generation nanotherapeutics medicine of extracellular vesicles. Int. J. Nanomedicine. 16, 3357–3383. 10.2147/IJN.S310357 34040369 PMC8140893

[B30] Huang L. Rong Y. Tang X. Yi K. Qi P. Hou J. (2022). Engineered exosomes as an *in situ* DC-primed vaccine to boost antitumor immunity in breast cancer. Mol. cancer 21 (1), 45. 10.1186/s12943-022-01515-x 35148751 PMC8831689

[B31] Hwang D. W. Choi H. Jang S. C. Yoo M. Y. Park J. Y. Choi N. E. (2015). Noninvasive imaging of radiolabeled exosome-mimetic nanovesicle using (99m). Tc-HMPAO. Scie. Rep. 5, 15636. 10.1038/srep15636 PMC4620485 26497063

[B32] Jun I. Choi W. Park M. (2018). Multi-block Analysis of genomic data using generalized canonical correlation analysis. Genomics & Inf. 16 (4), e33. 10.5808/GI.2018.16.4.e33 PMC6440675 30602094

[B33] Jung K. O. Kim Y. H. Chung S. J. Lee C. H. Rhee S. Pratx G. (2020). Identification of lymphatic and hematogenous routes of rapidly labeled radioactive and fluorescent exosomes through highly sensitive multimodal imaging. Int. J. Mol. Sci. 21 (21), 7850. 10.3390/ijms21217850 PMC7660226 33105908

[B34] Kamińska A. Marzec M. E. Stępień E. Ł. (2021). Design and optimization of a biosensor surface functionalization to effectively capture urinary extracellular vesicles. Mol. (Basel, Switz. 26 (16), 4764. 10.3390/molecules26164764 PMC8399133 34443351

[B35] Kamińska A. Roman M. Wróbel A. Gala-Błądzińska A. Małecki M. T. ProfPaluszkiewicz C. (2022). Raman spectroscopy of urinary extracellular vesicles to stratify patients with chronic kidney disease in type 2 diabetes. Nanomedicine Nanotechnol. Biol. Med. 39, 102468. 10.1016/j.nano.2021.102468 34619362

[B36] Karp J. S. Viswanath V. Geagan M. J. Muehllehner G. Pantel A. R. Parma M. J. (2020). PennPET explorer: Design and preliminary performance of a whole-body imager. J. Nucl. Med. 61 (1), 136–143. 10.2967/jnumed.119.229997 31227573 PMC6954465

[B37] Khan A. A. de Rosales R. T. M. (2021). Radiolabelling of extracellular vesicles for PET and SPECT imaging. Nanotheranostics 5 (3), 256–274. 10.7150/ntno.51676 33654653 PMC7914338

[B38] Khan A. A. Man F. Faruqu F. N. Kim J. Al-Salemee F. Carrascal-Miniño A. (2022). PET imaging of small extracellular vesicles via [89Zr]Zr(oxinate)4 direct radiolabeling. Bioconjug. Chem. 33 (3), 473–485. 10.1021/acs.bioconjchem.1c00597 35224973 PMC8931726

[B39] Kim D.-K. Lee J. Kim S. R. Choi D.-S. Yoon Y. J. Kim J. H. (2014). EVpedia: A community web portal for extracellular vesicles research. Bioinformatics 31 (6), 933–939. 10.1093/bioinformatics/btu741 25388151 PMC4375401

[B40] Kojima R. Bojar D. Rizzi G. Hamri G. C. El-Baba M. D. Saxena P. (2018). Designer exosomes produced by implanted cells intracerebrally deliver therapeutic cargo for Parkinson's disease treatment. Nat. Comm. 9 (1), 1305. 10.1038/s41467-018-03733-8 PMC5880805 29610454

[B41] Kooijmans S. Fliervoet L. van der Meel R. Fens M. Heijnen H. van Bergen En Henegouwen P. (2016). PEGylated and targeted extracellular vesicles display enhanced cell specificity and circulation time. J. Control Release. 224, 77–85. 10.1016/j.jconrel.2016.01.009 26773767

[B42] Krämer-Albers E. M. (2022). Extracellular vesicles at CNS barriers: Mode of action. Curr. Opin. Neurobiol. 75, 102569. 10.1016/j.conb.2022.102569 35667340

[B43] Królicki L. Kunikowska J. (2021). Theranostics - present and future. Bio-Algorithms Med-Systems. 17 (4), 213–220. 10.1515/bams-2021-0169

[B44] Kuska B. (1998). Beer, Bethesda, and biology: How "genomics" came into being. J. Natl. Cancer Inst. 90 (2), 93. 10.1093/jnci/90.2.93 9450566

[B45] Lai C. P. Mardini O. Ericsson M. Prabhakar S. Maguire C. Chen J. W. (2014). Dynamic biodistribution of extracellular vesicles *in vivo* using a multimodal imaging reporter. ACS Nano 8 (1), 483–494. 10.1021/nn404945r 24383518 PMC3934350

[B46] Lara P. Palma-Florez S. Salas-Huenuleo E. Polakovicova I. Guerrero S. Lobos-Gonzalez L. (2020). Gold nanoparticle based double-labeling of melanoma extracellular vesicles to determine the specificity of uptake by cells and preferential accumulation in small metastatic lung tumors. J. nanobiotechnology 18 (1), 20. 10.1186/s12951-020-0573-0 31973696 PMC6979068

[B47] Lázaro-Ibáñez E. Faruqu F. N. Saleh A. F. Silva A. M. Tzu-Wen Wang J. Rak J. (2021). Selection of fluorescent, bioluminescent, and radioactive tracers to accurately reflect extracellular vesicle biodistribution *in vivo* . ACS Nano 15 (2), 3212–3227. 10.1021/acsnano.0c09873 33470092 PMC7905875

[B48] Lee Y. J. Jy W. Horstman L. L. Janania J. Reyes Y. Kelley R. E. (1993). Elevated platelet microparticles in transient ischemic attacks, lacunar infarcts, and multiinfarct dementias. Thromb. Res. 72 (4), 295–304. 10.1016/0049-3848(93)90138-e 8303669

[B49] Li J. Komatsu H. Poku E. K. Olafsen T. Huang K. X. Huang L. A. (2022). Biodistribution of intra-arterial and intravenous delivery of human umbilical cord mesenchymal stem cell-derived extracellular vesicles in a rat model to guide delivery strategies for diabetes therapies. Pharm. (Basel, Switz. 15 (5), 595. 10.3390/ph15050595 PMC9143655 35631421

[B50] Liang T. Zhang R. Liu X. Ding Q. Wu S. Li C. (2021). Recent advances in macrophage-mediated drug delivery systems. Int. J. nanomedicine 16, 2703–2714. 10.2147/IJN.S298159 33854316 PMC8039204

[B51] Lind L. Kullberg J. Ahlström H. Michaëlsson K. Strand R. (2019). Proof of principle study of a detailed whole-body image analysis technique, “Imiomics”. regarding adipose lean tissue distribution. Sci. Rep. 9 (1), 7388. 10.1038/s41598-019-43690-w 31089168 PMC6517436

[B52] Lőrincz Á. M. Timár C. I. Marosvári K. A. Veres D. S. Otrokocsi L. Kittel Á. (2014). Effect of storage on physical and functional properties of extracellular vesicles derived from neutrophilic granulocytes. J. Extracell. Vesicles. 3, 25465. 10.3402/jev.v3.25465 25536933 PMC4275651

[B53] Lundström A. Mobarrez F. Rooth E. Thålin C. von Arbin M. Henriksson P. (2020). Prognostic value of circulating microvesicle subpopulations in ischemic stroke and TIA. Transl. Stroke Res. 11 (4), 708–719. 10.1007/s12975-019-00777-w 31983048 PMC7340656

[B55] Matsumoto A. Takahashi Y. Nishikawa M. Sano K. Morishita M. Charoenviriyakul C. (2017). Role of phosphatidylserine-derived negative surface charges in the recognition and uptake of intravenously injected B16bl6-derived exosomes by macrophages. J. Pharm. Sci. 106 (1), 168–175. 10.1016/j.xphs.2016.07.022 27649887

[B56] Matulewicz T. (2021). Radioactive nuclei for β+γ PET and theranostics: Selected candidates. Bio-Algorithms Med-Systems 17 (4), 235–239. 10.1515/bams-2021-0142

[B57] Möller A. Lobb R. J. (2020). The evolving translational potential of small extracellular vesicles in cancer. Nat. Rev. Cancer. 20 (12), 697–709. 10.1038/s41568-020-00299-w 32958932

[B58] Morishita M. Takahashi Y. Nishikawa M. Sano K. Kato K. Yamashita T. (2015). Quantitative analysis of tissue distribution of the B16BL6-derived exosomes using a streptavidin-lactadherin fusion protein and iodine-125-labeled biotin derivative after intravenous injection in mice. J. Pharm. Sci. 104 (2), 705–713. 10.1002/jps.24251 25393546

[B59] Moskal P. Dulski K. Chug N. Curceanu C. Czerwiński E. Dadgar M. (2021a). Positronium imaging with the novel multiphoton PET scanner. Sci. Adv. 7 (42), eabh4394. 10.1126/sciadv.abh4394 34644101 PMC11559468

[B60] Moskal P. Gajos A. Mohammed M. Chhokar J. Chug N. Curceanu C. (2021b). Testing CPT symmetry in ortho-positronium decays with positronium annihilation tomography. Nat. Commun. 12 (1), 5658. 10.1038/s41467-021-25905-9 34580294 PMC8476595

[B61] Moskal P. Kisielewska D. Y Shopa R. Bura Z. Chhokar J. Curceanu C. (2020). Performance assessment of the 2 γpositronium imaging with the total-body PET scanners. EJNMMI Phys. 7 (1), 44. 10.1186/s40658-020-00307-w 32607664 PMC7326848

[B62] Moskal P. Kowalski P. Shopa R. Y. Raczyński L. Baran J. Chug N. (2021c2019). Feasibility study of the positronium imaging with the J-PET tomographNEMA characteristics of the modular total-body J-PET scanner-an economic total-body PET from plastic scintillators. Phys. Med. Biol. Med. Biol. 6466 (517), 055017. 10.1088/1361-6560/aafe20lating10.1088/1361-6560/ac16bd10.1088/1361-6560/ac16bd(

[B63] Moskal P. Stępień E. Ł. (2020). Prospects and clinical perspectives of total-body PET imaging using plastic scintillators. Pet. Clin. 15 (4), 439–452. 10.1016/j.cpet.2020.06.009 32739047

[B64] Moskal P. Stępień E. (2022). Perspectives for translation of positronium imaging into clinics. Front. Phys. Sec. Med. Phys. Imaging 10, 969806. 10.3389/fphy.2022.969806

[B65] Murphy D. E. de Jong O. G. Brouwer M. Wood M. J. Lavieu G. Schiffelers R. M. (2019). Extracellular vesicle-based therapeutics: Natural versus engineered targeting and trafficking. Exp. Mol. Med. 51, 1–12. 10.1038/s12276-019-0223-5 PMC6418170 30872574

[B66] Németh K. Varga Z. Lenzinger D. Visnovitz T. Koncz A. Hegedűs N. (2021). Extracellular vesicle release and uptake by the liver under normo- and hyperlipidemia. Cell Mol. Life Sci. 78 (23), 7589–7604. 10.1007/s00018-021-03969-6 34665280 PMC8629784

[B67] Ohno S. Takanashi M. Sudo K. Ueda S. Ishikawa A. Matsuyama N. (2013). Systemically injected exosomes targeted to EGFR deliver antitumor microRNA to breast cancer cells. Mol. Ther. 21 (1), 185–191. 10.1038/mt.2012.180 23032975 PMC3538304

[B68] Opalińska M. Sowa-Staszczak A. Al Maraih I. Gilis-Januszewska A. Hubalewska-Dydejczyk A. (2021). Peptide receptor radionuclide therapy as a tool for the treatment of severe hypoglycemia in patients with primary inoperable insulinoma. Bio-Algorithms Med-Systems 17 (4), 221–226. 10.1515/bams-2021-0138

[B69] Paolini L. Federici S. Consoli G. Arceri D. Radeghieri A. Alessandri I. (2020). Fourier-transform Infrared (FT-IR) spectroscopy fingerprints subpopulations of extracellular vesicles of different sizes and cellular origin. J. Extracell. Vesicles. 9 (1), 1741174. 10.1080/20013078.2020.1741174 32341767 PMC7170381

[B70] Pierzchalski A. Mittag A. Tárnok A. (2011). Introduction A: Recent advances in cytometry instrumentation, probes, and methods--review. Methods Cell Biol. 102, 1–21. 10.1016/B978-0-12-374912-3.00001-8 21704833

[B71] Prenosil G. A. Sari H. Fürstner M. Afshar-Oromieh A. Shi K. Rominger A. (2022). Performance characteristics of the biograph vision quadra PET/CT system with a long axial field of view using the NEMA NU 2-2018 standard. J. Nucl. Med. 63 (3), 476–484. 10.2967/jnumed.121.261972 34301780

[B113] Roman M. Kamińska A. Drożdż A. Platt M. Kuźniewski M. Małecki M. T. (2019). Raman spectral signatures of urinary extracellular vesicles from diabetic patients and hyperglycemic endothelial cells as potential biomarkers in diabetes. Nanomedicine: Nanotech. Biol. Med. 17, 137–149. 10.1016/j.nano.2019.01.011 30703535

[B72] Royo F. Cossío U. Ruiz de Angulo A. Llop J. Falcon-Perez J. M. (2019). Modification of the glycosylation of extracellular vesicles alters their biodistribution in mice. Nanoscale 11 (4), 1531–1537. 10.1039/c8nr03900c 30623961

[B73] San Lucas F. A. Allenson K. Bernard V. Castillo J. Kim D. U. Ellis K. (2016). Minimally invasive genomic and transcriptomic profiling of visceral cancers by next-generation sequencing of circulating exosomes. Ann. Oncol. 27 (4), 635–641. 10.1093/annonc/mdv604 26681674 PMC4803451

[B74] Shan S. Chen J. Sun Y. Wang Y. Xia B. Tan H. (2022). Functionalized macrophage exosomes with panobinostat and ppm1d-siRNA for diffuse intrinsic pontine gliomas therapy. Adv. Sci. (Weinheim, Baden-Wurttemberg, Ger. 9 (21), e2200353. 10.1002/advs.202200353 PMC9313473 35585670

[B75] Shi S. Li T. Wen X. Wu S. Y. Xiong C. Zhao J. (2019). Copper-64 labeled PEGylated exosomes for *in vivo* positron emission tomography and enhanced tumor retention. Biocon. Chem. 30 (10), 2675–2683. 10.1021/acs.bioconjchem.9b00587 PMC6947533 31560538

[B76] Skotland T. Ekroos K. Kauhanen D. Simolin H. Seierstad T. Berge V. (2017). Molecular lipid species in urinary exosomes as potential prostate cancer biomarkers. Eur. J. Cancer. 70, 122–132. 10.1016/j.ejca.2016.10.011 27914242

[B77] Skotland T. Iversen T. G. Llorente A. Sandvig K. (2022). Biodistribution, pharmacokinetics and excretion studies of intravenously injected nanoparticles and extracellular vesicles: Possibilities and challenges. Adv. Drug Deliv. Rev. 186, 114326. 10.1016/j.addr.2022.114326 35588953

[B78] Skotland T. Sagini K. Sandvig K. Llorente A. (2020). An emerging focus on lipids in extracellular vesicles. Adv. Drug Deliv. Rev. 159, 308–321. 10.1016/j.addr.2020.03.002 32151658

[B79] Son S. H. Oh J. M. Gangadaran P. Ji H. D. Lee H. W. Rajendran R. L. (2020). White blood cell labeling with Technetium-99m (99mTc) using red blood cell extracellular vesicles-mimetics. Blood cells, Mol. Dis. 80, 102375. 10.1016/j.bcmd.2019.102375 31655394

[B80] Spencer B. A. Berg E. Schmall J. P. Omidvari N. Leung E. K. Abdelhafez Y. G. (2021). Performance evaluation of the uEXPLORER total-body PET/CT scanner based on NEMA NU 2-2018 with additional tests to characterize PET scanners with a long axial field of view. J. Nucl. Med. official Publ. Soc. Nucl. Med. 62 (6), 861–870. 10.2967/jnumed.120.250597 PMC8729871 33008932

[B81] Stahlschmidt R. S. Ulfenborg B. Synnergren J. (2022). Multimodal deep learning for biomedical data fusion: A review. Briefings Bioinforma. 23 (2), bbab569. 10.1093/bib/bbab569 PMC8921642 35089332

[B82] Stępień E. Kamińska A. Roman M. Paluszkiewicz C. (2020). Method of detecting and diagnosing the course of diabetes. PL Patent No 235682 Patent office of the Republic of Poland (UPRP).

[B83] Stępień E. Ł. Durak-Kozica M. Kamińska A. Targosz-Korecka M. Libera M. Tylko G. (2018). Circulating ectosomes: Determination of angiogenic microRNAs in type 2 diabetes. Theranostics 8 (14), 3874–3890. 10.7150/thno.23334 30083267 PMC6071541

[B84] Stępień E. Ł. Kamińska A. Surman M. Karbowska D. Wróbel A. Przybyło M. (2021a). Fourier-Transform InfraRed (FT-IR) spectroscopy to show alterations in molecular composition of EV subpopulations from melanoma cell lines in different malignancy. Biochem. Biophys. Rep. 25, 100888. 10.1016/j.bbrep.2020.100888 33458258 PMC7797365

[B85] Stępień E. Ł. Rząca C. Moskal P. (2021b). Novel biomarker and drug delivery systems for theranostics – extracellular vesicles. Bio-Algorithms Med-Systems. 17 (4), 301–309. 10.1515/bams-2021-0183

[B86] Stępień E. Stankiewicz E. Zalewski J. Godlewski J. Zmudka K. Wybrańska I. (2012). Number of microparticles generated during acute myocardial infarction and stable angina correlates with platelet activation. Arch. Med. Res. 43 (1), 31–35. 10.1016/j.arcmed.2012.01.006 22306248

[B87] Strand R. Malmberg F. Johansson L. Lind L. Sundbom M. Ahlström H. (2017). A concept for holistic whole body MRI data analysis, Imiomics. PLOS ONE 12 (2), e0169966. 10.1371/journal.pone.0169966 28241015 PMC5328256

[B88] Surman M. Hoja-Łukowicz D. Szwed S. Drożdż A. Stępień E. Przybyło M. (2018). Human melanoma-derived ectosomes are enriched with specific glycan epitopes. Life Sci. 207, 395–411. 10.1016/j.lfs.2018.06.026 29959030

[B89] Sutaria D. S. Jiang J. Elgamal O. A. Pomeroy S. M. Badawi M. Zhu X. (2017). Low active loading of cargo into engineered extracellular vesicles results in inefficient miRNA mimic delivery. J. Extracell. Vesicles. 6 (1), 1333882. 10.1080/20013078.2017.1333882 28717424 PMC5505005

[B90] Tataruch-Weinert D. Musante L. Kretz O. Holthofer H. (2016). Urinary extracellular vesicles for RNA extraction: Optimization of a protocol devoid of prokaryote contamination. J. Extracell. Vesicles. 5, 30281. 10.3402/jev.v5.30281 27345058 PMC4921785

[B91] Théry C. Witwer K. W. Aikawa E. Alcaraz M. J. Anderson J. D. Andriantsitohaina R. (2018). Minimal information for studies of extracellular vesicles 2018 (MISEV2018): A position statement of the international society for extracellular vesicles and update of the MISEV2014 guidelines. J. Extracell. Vesicles. 7 (1), 1535750. 10.1080/20013078.2018.1535750 30637094 PMC6322352

[B92] Tokarz A. Konkolewska M. Kuśnierz-Cabala B. Maziarz B. Hanarz P. Żurakowski A. (2019). Retinopathy severity correlates with RANTES concentrations and CCR 5-positive microvesicles in diabetes. Folia Med. cracov. 59 (3), 95–112. 10.24425/fmc.2019.131139 31891363

[B93] Tokarz A. Szuścik I. Kuśnierz-Cabala B. Kapusta M. Konkolewska M. Żurakowski A. (2015). Extracellular vesicles participate in the transport of cytokines and angiogenic factors in diabetic patients with ocular complications. Folia Med. cracov. 55 (4), 35–48. 26867118

[B94] Turek C. Wróbel S. Piwowar M. (2020). OmicsON - integration of omics data with molecular networks and statistical procedures. PloS one 15 (7), e0235398. 10.1371/journal.pone.0235398 32726348 PMC7390260

[B95] Valet G. (2022). Cytomics. Avcaliable at: https://www.classimed.de/cytomics.html#cytomics .

[B96] Valet G. Murphy R. F. Robinson J. P. Tarnok A. Kriete A. (2006). “Cytomics: From cell States to predictive medicine,” in Computational systems biology (Elsevier), 363–381. 10.1016/b978-012088786-6/50035-6

[B97] van der Pol E. Coumans F. Varga Z. Krumrey M. Nieuwland R. (2013). Innovation in detection of microparticles and exosomes. J. Thromb. Haemost. 11 (1), 36–45. 10.1111/jth.12254 23809109

[B98] van Dommelen S. M. Vader P. Lakhal S. Kooijmans S. A. van Solinge W. W. Wood M. J. (2012). Microvesicles and exosomes: Opportunities for cell-derived membrane vesicles in drug delivery. J. Contr. Release 161 (2), 635–644. 10.1016/j.jconrel.2011.11.021 22138068

[B99] Vandenberghe S. Moskal P. Karp J. S. (2020). State of the art in total body PET. EJNMMI Phys. 7, 35. 10.1186/s40658-020-00290-2 32451783 PMC7248164

[B100] Wang N. Chen C. Yang D. Liao Q. Luo H. Wang X. (2017). Mesenchymal stem cells-derived extracellular vesicles, via miR-210, improve infarcted cardiac function by promotion of angiogenesis. Biochimica Biophysica Acta (BBA) - Mol. Basis Dis. 8, 2085–2092. 10.1016/j.bbadis.2017.02.023 28249798

[B101] Wang S. E. (2022). Extracellular vesicles in cancer therapy. Sem. Cancer Biol. S1044-579X(22)00135-3 Advance online publication. 10.1016/j.semcancer.2022.06.001 PMC10431950 35688334

[B102] Weineisen M. Schottelius M. Simecek J. Baum R. P. Yildiz A. Beykan S. (2015). 68Ga- and 177Lu-labeled PSMA I&T: Optimization of a PSMA-targeted theranostic concept and first proof-of-concept human studies. J. Nucl. Med. 56 (8), 1169–1176. 10.2967/jnumed.115.158550 26089548

[B103] Wiklander O. P. Nordin J. Z. O'Loughlin A. Gustafsson Y. Corso G. Mäger I. (2015). Extracellular vesicle *in vivo* biodistribution is determined by cell source, route of administration and targeting. J. Extracell. Vesicles. 4, 26316. 10.3402/jev.v4.26316 25899407 PMC4405624

[B104] Williams C. Royoa F. Aizpurua-Olaizola O. Pazosb R. Boons G.-J. Reichardt N.-C. (2018). Glycosylation of extracellular vesicles: Current knowledge, tools and clinical perspectives. J. Extracell. Vesicles. 7 (1), 1442985. 10.1080/20013078.2018.1442985 29535851 PMC5844028

[B105] Wróbel S. (2021). Canonical correlation analysis of m data obtained from microvesicles of human skin melanoma cell lines and its possible application in biomarker discovery. Cracow: Jagiellonian University. PhD Thesis.

[B106] Wu J. Y. Li Y. J. Hu X. B. Huang S. Xiang D. X. (2021). Preservation of small extracellular vesicles for functional analysis and therapeutic applications: A comparative evaluation of storage conditions. Drug Deliv. 28 (1), 162–170. 10.1080/10717544.2020.1869866 33427518 PMC7808382

[B107] Yadav S. P. (2007). The wholeness in suffix -omics, -omes, and the word om. J. Biomol. Tech. JBT 18 (5), 277. 18166670 PMC2392988

[B108] Zhu Z. Zhai Y. Hao Y. Wang Q. Han F. Zheng W. (2022). Specific anti-glioma targeted-delivery strategy of engineered small extracellular vesicles dual-functionalised by Angiopep-2 and TAT peptides. J. Extracell. Vesicles. 11 (8), e12255. 10.1002/jev2.12255 35932288 PMC9451528

